# The Dynamics of Urban Ecosystem Governance in Rotterdam, The Netherlands

**DOI:** 10.1007/s13280-014-0512-0

**Published:** 2014-04-17

**Authors:** Niki Frantzeskaki, Nico Tilie

**Affiliations:** 1 Faculty of Social Sciences, Dutch Research Institute for Transitions (DRIFT), Building T16 Room 42, PO Box 1738, 3000 DR Rotterdam, The Netherlands; 2 Erasmus University Rotterdam, Rotterdam, The Netherlands; 3 Department of Urbanism, Faculty of Architecture, Delft University of Technology, Julianalaan 134, 2628 BL Delft, The Netherlands

**Keywords:** Urban delta, Governance, Urban ecosystems, Ecosystem services, Densification

## Abstract

We explore whether Rotterdam city has the governance capacity in terms of processes at place, and the attention in terms of vision and strategy to take up an integrated approach toward urban resilience. We adopt an interpretative policy analysis approach to assess the dynamics of urban ecosystem governance considering interviews, gray literature, and facilitated dialogues with policy practitioners. We show the inner workings of local government across strategic, operational, tactical, and reflective governance processes about the way urban ecosystems are regulated. Despite the existing capacity to steer such processes, a number of underlying challenges exist: need for coordination between planning departments; need to ease the integration of new policy objectives into established adaptive policy cycles; and need to assess the lessons learnt from pilots and emerging green initiatives. Regulating and provisioning ecosystem services receive heightened policy attention. Focus on regulating services is maintained by a policy renewal cycle that limits and delays consideration of other ecosystem services in policy and planning.

**Electronic supplementary material:**

The online version of this article (doi:10.1007/s13280-014-0512-0) contains supplementary material, which is available to authorized users.

## Introduction

The mounting climate change pressures to cities, in synergy with social dynamics, globalization, and urbanization, create new challenges that require cities to act upon them differently (Dobbelsteen et al. 2010). The growth of modern cities results in ever larger dependence on hinterlands for their surviving (Brand and Vadrot 2013). Cities as cradles of socio-technological change and innovation could build engineered resilience that make them habitable. Urban delta cities are examples of the way human ingenuity and engineered solutions allow settlement in flood-prone areas. The solely technocratic solutions from the past seem to be limited in the solutions they can offer, and the diversity of needs of many inhabitants can no longer be served. The urban quest has turned from “creating a habitable space” to “creating a resilient and livable place to live, work and play” (Tillie et al. 2012; Ferguson et al. 2013; Jim 2013). Holistic approaches, that consider the multiple functions of urban space, are required for a sustainability transition that develops a new urban social–ecological contract (Ness et al. 2007; Tanguay et al. 2010).

Integrated approaches to urban governance have been advocated by urban planners and urban planning scholars alike as desirable for bringing sustainability agendas into practice (Taylor et al. 2012; Wilkinson et al. 2013). Conceptualizing cities as social–ecological systems suggests that integrative frameworks are needed to aid cities to their transition to urban resilience (Ernstson et al. 2010; Jansson 2013; Frantzeskaki et al. 2014). The adoption of new scientific frameworks from policy and planning is not easy: existing structures of policy processes, dominant discourses, and entrenched policy practices for designing and adapting policies may create non-hospitable contexts for new frameworks to be considered and/or adopted.

We explore whether Rotterdam city has the governance capacity in terms of processes at place, and the spectrum of attention in terms of vision and strategy to take up a integrated approach toward urban resilience. With urban ecosystem governance we conceptualize the processes and practices steered by agencies that manage, draw policy and plans, as well as regulate and monitor the conservation, maintenance, and restoration natural capital in an urban context. Our paper focuses on the following research question: What are the governance processes and practices for urban ecosystems governance in Rotterdam city in view of its quest for urban sustainability and resilience?

### Research Approach

Given that our research is qualitative, we have used three phases to triangulate our findings: (a) Data collection, including gray literature such as plans, policies, and visions of the Rotterdam city and in-person interviews with planners, practitioners, and experts; (b) Data analysis, including a governance context analysis and ecosystem services mapping in existing policy and planning documents (TEEB 2011); and (c) Data validation, realized by facilitated and planned participatory sessions with stakeholders. A detailed description of every phase including the specific research methods is provided in Electronic Supplementary Material. In the following paragraphs we present the conceptual frameworks used to analyze our data and address the research question.

### Conceptual Framework

#### Multi-Level Governance: Actions and Processes

The governance context will be analyzed by applying the multi-level governance framework that focuses on activities and processes across four levels of governance organization: (a) strategic level including processes and activities of setting long-term goals, policy development, planning, vision, values, identity, and culture of the city; (b) tactical level including designing steering activities, programs, funding (Loorbach 2010; Frantzeskaki et al. 2014), and establishment of networks and/or partnerships; (c) operational level including implementing and managing policy action plans, infrastructure plans and assets; and (d) reflexive level with monitoring and evaluating existing policies and assets and their interaction with citizens. For assessing the type of activities (strategic, tactical, operational, and reflexive) we need to understand and map the relations between different local government departments within and across the multiple governance levels.

#### Urban Ecosystem Services: From Scientific Assessment to Policy Diagnostics

For examining the governance attention to ecosystem services as conditions to “achieve” an integrated approach to urban ecosystem governance, we apply the TEEB (2011) frame of ecosystem services. The ecosystem services frame identifies four types of services ecosystems provide to humans (cf. Millenium Ecosystem Assessment): Supporting (that relate to habitat), provisioning (that relate to yields directly harvested by humans), regulating (that relate to indirect benefits by ecosystems), and cultural services (that relate to intangible benefits).

Recent research focuses on extending the application of ecosystem services framework to the urban context (Jansson 2013). The value of ecosystem services in environmental policy design processes in cities has been researched by recent scholarship addressing it as a policy analysis and design tool (Menzel and Teng 2010; Hauck et al. 2013; Wilkinson et al. 2013) as a diagnostic tool (Cowling et al. 2008; Daily et al. 2009; Vihervaara et al. 2010) or a policy evaluation tool (de Groot et al. 2010; Sijtsma et al. 2013). The ecosystem services framework may serve as a frame for discovering opportunities to restore ecosystems when understanding cities as socio-ecological systems (Nicholson-Lord 1987; Beatley 2011).

In their critical analysis on the applications of the ecosystem services framework for planning, Primer and Furman (2012, p. 86) note that the missing step is the understanding of the governance context of application: “Although the ecosystem service approaches often pay attention to social-ecological systems and allow considering a range of issues simultaneously, they do not provide direct solutions to ecosystem service governance because they do not take existing administrative and governance structures and practices as a starting point.” From the applications of ecosystem services to explore urban governance, Hauck et al. (2013, p. 20) addressed that ecosystem services may be a comprehensive framework for environmental benefits but “the concept of ecosystem services lends itself to oversimplification on a higher level while situations on regional and local level are more complex.” Wilkinson et al. (2013) argue that “even in its most basic form the ecosystem services framework is a useful policy analysis tool to expose the specific way in which ecosystem related matters are addressed in the strategic spatial planning policy discourse. Importantly it also reveals which ecosystem services are left out of the discourse.” Following this, we apply the ecosystem services framework to explore the attention in urban governance for ecosystems’ conservation and restoration.

## Urban Ecosystems and Their Governance in Rotterdam

In this section, first we present an overview of the existing urban ecosystems of Rotterdam city, second we present the organizational structure of the city’s administration, and third we zoom in the different governance levels to examine the dynamics of urban ecosystem governance.

### Urban Ecosystems of Rotterdam City

**Fig. 1:**
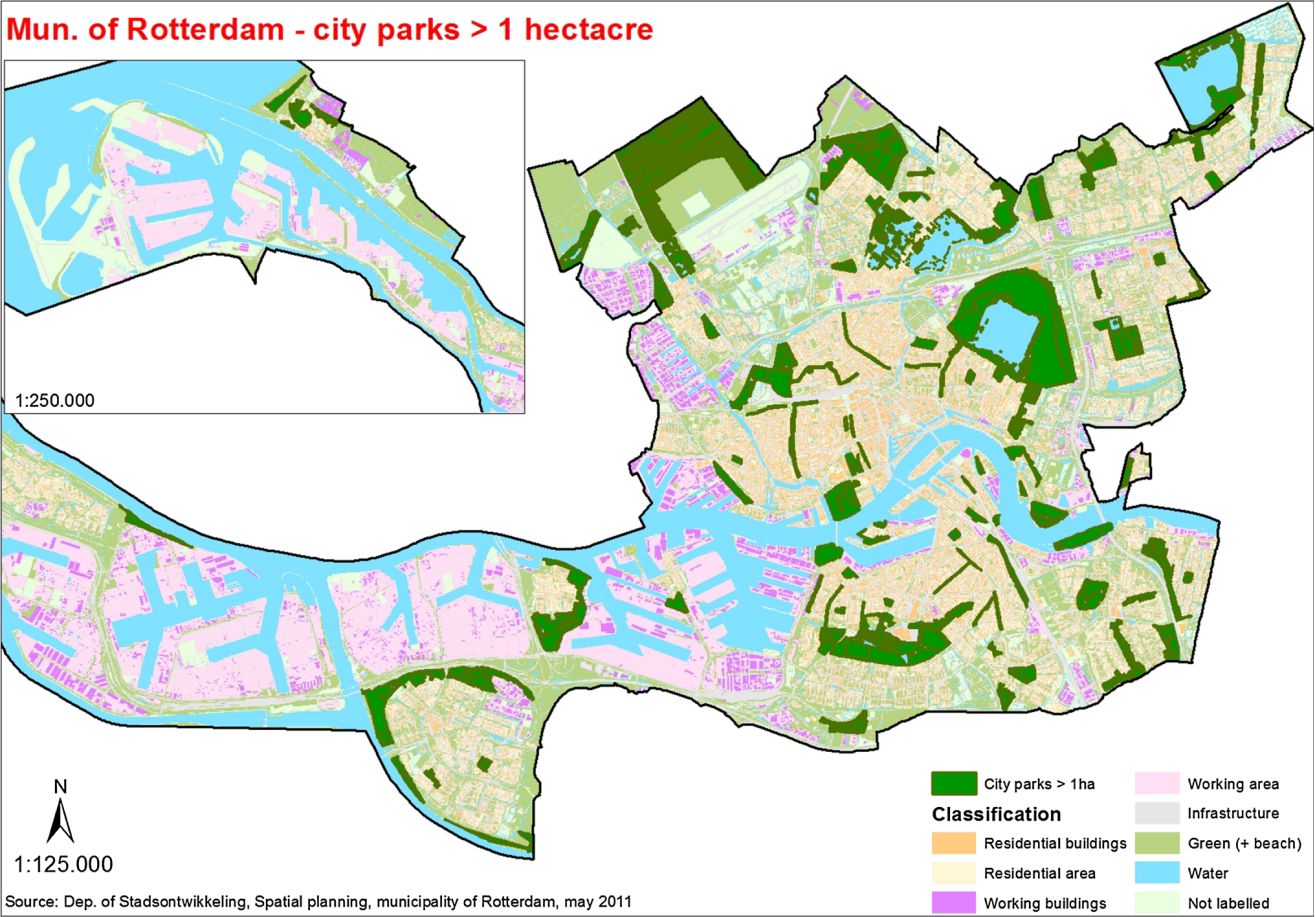
Land-use map of the City of Rotterdam (*Source* Municipality of Rotterdam, May 2011)

Rotterdam is located in the Rhine–Meuse Delta and accommodates 611 000 people of no less than 173 different nationalities. Rotterdam is the largest port in Europe and the lowest delta in Europe. Rotterdam faces climatic uncertainties and pressures. Rotterdam is at the same time one of the greenest large cities of the Netherlands. A total of 747 000 trees grow in the parks, port, and alongside the rivers. It has a total of 117 public parks (1765 ha), some of which are well known like Zuiderpark and Kralingse Bos (Fig. 1). Green space in Rotterdam covers 19.7 % of the total city’s surface whereas water amounts to a 34.9 % including the harbor (Gemeente Rotterdam 2010).

### The Current Organizational Architecture

During the scoping phase of the research we found that there are three municipality offices responsible for urban ecosystem governance in the City of Rotterdam:

*Urban Development Office* (Stadsontwikkeling in Dutch) is responsible for formulation and implementation of vision, strategy, and policy programs for the urban environment. It deals with the maintenance, designation, and restoration of urban characteristics such as green space, parks, streetscapes, and waterscapes of the city and of all other cityscape facilities (e.g., street furniture). This office oversees the implementation of urban development programs and plans.

*Sustainability Planning Office* is responsible for the formulation of vision, strategy, and programs for sustainability as an overarching theme that merges energy planning, air quality planning, and noise regulation planning. The sustainability planning office is responsible to inform and advise the urban development office and the relating climate adaptation office.

*Climate Adaptation Office* is responsible for strategic advice and strategy formulation for protecting Rotterdam city from climate change impacts. It is also responsible for keeping climate adaptation plans updated via ensuring consideration of new scientific knowledge about climate change impacts. The Climate Adaptation Office deals with issues on coastal water management of the city.

There are, however, two organizations that in addition to the Climate Adaptation Office provide advice about climate change adaptation targets and measures: the *Rotterdam Climate Initiative* and the *Delta Program*. The Rotterdam Climate Initiative includes a variety of actors from the local level and it is a platform for horizontal integration. The Delta Program is a platform for policy implementation that integrates and bridges national, regional, and local administrative and advisory levels. Both bridging networks formulated visions, programs, and measures that refer to urban ecosystems as a result of a successful cooperation with the Municipality of Rotterdam.

The *Rotterdam Climate Initiative* is a learning platform of the Rotterdam Municipality, the Port Authorities, Deltalings and the environmental protection agency founded in 2007. The Rotterdam Climate Initiative acts as knowledge liaison with the objectives to disseminate knowledge between the partners in order to create solutions that can contribute to achieving the agreed upon target of 50 % reduction in CO_2_ emissions in harbor and city in 2025 taking 1990 as the reference year. The second target concerns making Rotterdam Climate Proof against flooding and urban heat island effect in 2025. Knowledge and analysis from the Rotterdam Climate Initiative have informed the sustainability vision and the urban planning programs since 2010 (Gemeente Rotterdam 2012a).

The *Delta Program* is the policy implementation platform of the new National Water Plan of the Netherlands. The main target of the Delta Program is to ensure safety from floods while guaranteeing the supply of fresh water. It consists of three regional platforms one of which includes the city of Rotterdam. The Delta Program is a platform for vertical integration since it involves the national government, provinces, water authorities, and municipalities and actively engages with community and research. The regional Delta Program for the case of Rotterdam highlights vulnerabilities and opportunities that relate to flood risks and to existing measures for flood defenses. Delta Program has a very long-term horizon (2100) and seeks for integral measures to safeguard Rotterdam from future climate vulnerabilities. The flood defense measures for Rotterdam have been designed in a collaborative process between the Urban Development Office and the Ministry of Infrastructures and Environment that oversees the design and implementation of the Delta Program at the national level. For the implementation of the regional Delta Program the interviewees considered as the main challenge the mismatch of time scales: Delta Program has a long-term horizon (2100) whereas urban planning programs have shorter term horizons. This mismatch generates new uncertainties.

### Governance Dynamics Across Levels

Policy and planning institutions in Rotterdam are well equipped with knowledge and expertise to develop policy plans and implement them in an effective way. Over the years, numerous research-policy projects and partnerships have been established that resulted in an increasing knowledge capacity and expertise of city planners (see Frantzeskaki et al. 2014). This, however, does not imply a flawless policy and planning structure to deal with the complex issues of urban green and blue infrastructure. In the transition of the city to modern governance, multiple processes and ambitions create systemic complexity. The identified challenges represent the current complexity of the governance context in Rotterdam city and are considered important to understand and tackle in order to implement integrated plans that promote urban sustainability and resilience. The underlying challenges that relate to the way ecological features in Rotterdam city are planned and managed are interrelated and lie at four governance levels: strategic, operational, tactical, and reflexive. In Table 1 we present an overview of the underlying challenges and in Table 2 we present statements from practitioners that explicate their understanding of these governance challenges.

**Table 1:** Underlying challenges across governance levels

Challenges at the strategic governance level
(a) Policy attention converged toward densification of the inner city and maintenance of existing green spaces
(b) Current strategy of densification may limit opportunities for greening in the inner city area of Rotterdam whereas space for experimenting may be freed up in the periphery of the city
(c) The majority of the well-structured and elaborate strategic plans and visions zoom in the inner city and there is a lack of an overarching city-wide vision and strategy for urban ecosystems and their governance
Challenges at the tactical governance level
(a) Green and blue areas are considered as distinct rather than interdependent urban ecosystems
(b) Lack of a holistic approach to consider all aspects of urban ecosystems and environmental quality at city scale
(c) Disconnection between long-term vision and short-term and medium-term action in planning programs
(d) Current ways of engaging with citizens have to be updated to fit new social dynamics and needs
Challenges at the operational governance level
(a) Synergies between planned (before putting on implementation) and ongoing measures are not exploited due to limited information sharing and coordination
(b) There is a need for planning guidelines to designate green areas based on the benefits that can be received from the different types of green
(c) There is no effective strategy on how to scale successful examples of greening in Rotterdam to other locations in the city
Challenges at the reflexive governance level
(a) During the evaluation of implemented strategies, strategies that deliver multiple benefits are not examined
(b) There is limited assessment of social dynamics and needs in the way they are depicted in strategic objectives and targets

**Table 2:** Understanding of governance challenges as expressed by planners and practitioners

Governance level	Identified challenges	Expressed understanding of challenges by practitioners and planners
Strategic	Strategic plans and visions zoom in the inner city and there is a lack of an overarching city-scale vision	There is lack of city-scale understanding of challenges and this is also the reason of not having a new city-wide vision after the Urban Vision of 2005 Attempts to (have a visionary) plan at city-scale have failed
Tactical	Green and blue areas are considered as distinct rather than interdependent urban ecosystems	The fragmentation of the Rotterdam Municipality’s organizational structure is a disturbing factor for having an overarching approach, vision and plan There is a challenge on center-staging the river as a carrier for biodiversity and other recreational activities
	Lack of a holistic approach to consider all aspects of urban ecosystems	(we) see ecology as an aspect on the checklist for planning that needs to be ticked off rather than as an integral part of the planning and designing process (we) need to seize the opportunity to link the outer space (green space) agenda with other policy agendas such as health agenda
	Disconnection between long-term vision and short-term and medium-term action in planning programs	The difference between time scales creates opportunities and uncertainties. Urban planning actions focus on short-term, are more operational
	Current ways of engaging with citizens have to be updated to fit new social dynamics and needs	There is the need for more knowledge and consultation with citizens and practitioners at early stages of policy development It is a challenge for Rotterdam to engage with people from different ethnic and cultural backgrounds when considering ecosystems’ conservation and restoration given the different take they may have on biodiversity We may need to think of giving power to citizens about the design and maintenance of green public space
Operational	Synergies between planned and ongoing measures are not exploited	On certain programs and projects (e.g. green roofs) inter-organizational networks are established, but this is more incidental than structural What is missing is the integration of the green strategy with aspects of biodiversity
	Need for planning guidelines to inform designation areas for green	There is a challenge where to find space to place green and especially trees. Shall we consider new types of green like on roofs, temporary green or trees-on-wheels? From a health’s perspective, it can be advocated that adding more green spaces to the mix of plans and measures will benefit urban citizens’ health, but from climate adaptation point of view alone, there is no need for more green but an examination of the benefits that additional green space can bring in
	No effective strategy on how to scale successful examples of greening	(we) need to think which pilots we can replicate but there is no vision, no strategy about it
Reflexive	Need for new evaluation methods of implemented policies	There is a need to design plans and policies based on functionality and demand (…) rather than comparing future plans with current situation

#### Strategic Governance Level

The strategic governance level maps processes and activities of setting long-term goals, policy development processes, planning, creating visions, values, identity, and culture of the city. The underlying challenges at the strategic governance level that relate to the way ecological features in Rotterdam city are planned pointed at the following interrelated challenges: (a) The policy attention, across the visions and strategic plans and programs, is on establishing an identity of the city via ensuring livability, financial strength and continuity, as well as adaptation and protection from climate pressures. Over the years, the visions converged toward densification and maintenance of existing green spaces; (b) The densification strategic pathway is envisioned to work in partnership with greening strategic pathway but the realization of these two pathways includes persistent trade-offs; (c) The majority of the well-structured and elaborate strategic plans and visions zoom in the inner city and there is a lack of an overarching citywide vision and strategy for urban ecosystems and their governance. The above presented challenges will be further elaborated below.

(a) *Policy attention converged toward densification of the inner city and maintenance of existing green spaces.* The Green Strategy proposes the establishment of two co-centric green rings that will be cross-linked with the blue corridors that rivers Rotte and Schie create (Gemeente Rotterdam 2005). The green strategy prioritizes the connections between existing and future green areas. Its implementation resulted in more than 500 hectares of new green space that is now used for recreation and is accessible via 13 km of cycle paths. However, since 2005 there has been no urban green vision at city scale since the focus shifted to the city center’s restoration and improvement.

The Gemeente Rotterdam (2007a) contains the spatial development strategy until 2030 and is the point of departure for quality improvement on multiple domains such as public space, sustainability, housing, and accessibility. The spatial development strategy builds on the understanding that different domains within the city are interdependent and these interdependencies need to be considered when designing interventions at all city scales. In the Rotterdam Urban Vision, we observe an anthropocentric focus on the socio-cultural dynamics and how to enhance and sustain them. A key decision is to meet housing demand by building within the existing urban area. The densification strategy becomes a prevailing solution given the merits it shows in preserving ecosystems and green areas located in the peri-urban area. This is not, however, how the densification strategy received increased attention at present.

The Gemeente Rotterdam (2007a) was followed by the vision for urban structure (2009a). The vision for urban structure (Gemeente Rotterdam (2009) following the Handbook of Rotterdam’s Style, 2008, *de Rotterdamse Stijl* in Dutch) sets the benchmark for what can be introduced in Rotterdam city in terms of urban design and also positions green infrastructure as an integral element of city’s structure. The green infrastructure is addressed to provide benefits such as recreation, amenity, aesthetic appreciation, sense of place and time (by estimating and indicating where trees tend to root and grow strong and by suggesting letting trees grow old in the city), and maintaining the diversity of the city’s plants and trees. This aspect comes with an argumentation of the trees’ aesthetic appeal rather than about the tree species diversity and the associated ecosystem services. In this vision there is a quota on how existing green spaces need to be maintained so as to ensure good quality and their sustainability.

Introducing more green pockets in the city is prioritized by the Sustainability Program. In the Sustainability program, greening of the city is referred to contribute to climate regulation, to regulating heat stress, to improve health, and to buffer industrial noise. This is the first program that addresses the many benefits of green spaces for citizens and explicitly shows its multivariate contribution to urban sustainability (Gemeente Rotterdam 2012a).

The Green Program for the inner city proposed in 2013 specifies that the strategies on greening aim at improving the quality of green space, the reestablishment of green in neighborhoods that are impoverished of green space, and the maintenance of tree diversity in the city (Gemeente Rotterdam 2013). Albeit not explicitly addressed in the Green Program, the strategies for inner city greening do agree with the proposed Gemeente Rotterdam (2005) and with the guidelines of the spatial strategy of the Gemeente Rotterdam (2007a). The Green Program also includes a strategy about the use of the waterscape for recreation.

Densification strategy becomes an alternative of the Sustainability Vision presented at the 5th International Architecture Biennale Rotterdam (Tillie et al. 2012). In the sustainability vision, two processes are conceptualized to contribute to sustainability: densification and greening of the inner city. The anthropocentric focus of the Sustainability Vision is supported by the scope of these two strategic objectives: to ensure energy safety of the city without compromising its livability. In this strategic plan, densification is proposed as a solution that can benefit the city by providing more jobs, improving its energy efficiency, and downscaling its energy footprint.

(b) *Current strategy of densification may limit opportunities for greening in the inner city area of Rotterdam whereas space for experimenting may be freed up in the periphery of the city.* Densification of the inner city area is the planning strategy for proactively designing how to accommodate more housing demand in the city of Rotterdam so as to ensure that existing green areas will be conserved. Densification in areas of medium-density infill can limit the possibilities for greening in areas of the inner city that are now viewed as potential sites for green restoration. From the planning scholarship, it is argued that dense cities offer more possibilities for urban biodiversity if the densification comes in concert with natural retrofitting (Beatley 2011, pp. 152–153). This, however, is not recognized as a trade-off by the Sustainability Vision (Gemeente Rotterdam 2012a). On the contrary, it is proposed that “smart densification must go hand in hand with the qualitative upgrading and quantitative expansion of urban green” (Tillie et al. 2012, p. 11). Densification in this way limits the introduction of different greening actions such as a better green network for biking and walking; turning parking lots into waterfront parks; and using wide green walls, green roofs, and facades in existing buildings. The Sustainability Vision calls for integrating more green into the inner city. However, except from turning parking lots and areas near the waterfront into green spaces. There is no reference for creating new green space at the surface that can be used as public green space. To conclude, even though the Sustainability Vision offers a view on how to better integrate solutions to meet the needs for more housing and more green space in the inner city, it points out that there is a trade-off in achieving these needs.

(c) *The majority of the well*-*structured and elaborate strategic plans and visions zoom in the inner city whereas there is a lack of an overarching city*-*wide vision and strategy for urban ecosystems and their governance.* The majority of the visions focus on the inner city or the city center (Gemeente Rotterdam 2007b, 2012b; Tillie et al. 2012). The Gemeente Rotterdam (2005) proposed measures for green and blue networks at a city scale but it is just one of the few strategic plans that addresses connectivity of green networks at that scale.

#### Tactical Governance Level

The tactical governance level maps the designing of steering activities, programs, funding, and establishment of networks and/or partnerships. On this level, we position the planning approach and its associated steering activities and programs. The *underlying challenges at the tactical governance level that relate to the way ecological features in Rotterdam city are planned* pointed at the following interrelated challenges: (a) Green and blue areas are considered as distinct rather than interdependent urban ecosystems; (b) Lack of a holistic approach to consider all aspects of urban ecosystems and environmental quality at city scale; (c) Disconnection between long-term vision and short-term and medium-term action in planning programs; and (d) Current ways of engaging with citizens have to be updated to fit new social dynamics and needs. The above challenges will be further elaborated below.

(a) *The current planning approach has treated green and blue areas mainly as recreational areas and built elements and not as urban ecosystems. This results in a disintegrated approach that considers green and blue areas as distinct rather than interdependent urban ecosystems*. The current planning approach of blue infrastructure has focused on technical functionality for drainage, use for shipping, and recreation rather than connecting the water scape with green spaces and seeing to its provisioning of multiple benefits. This is due to the legacy of reliable infrastructure planning in Rotterdam and in the Netherlands overall.

**Fig. 2:**
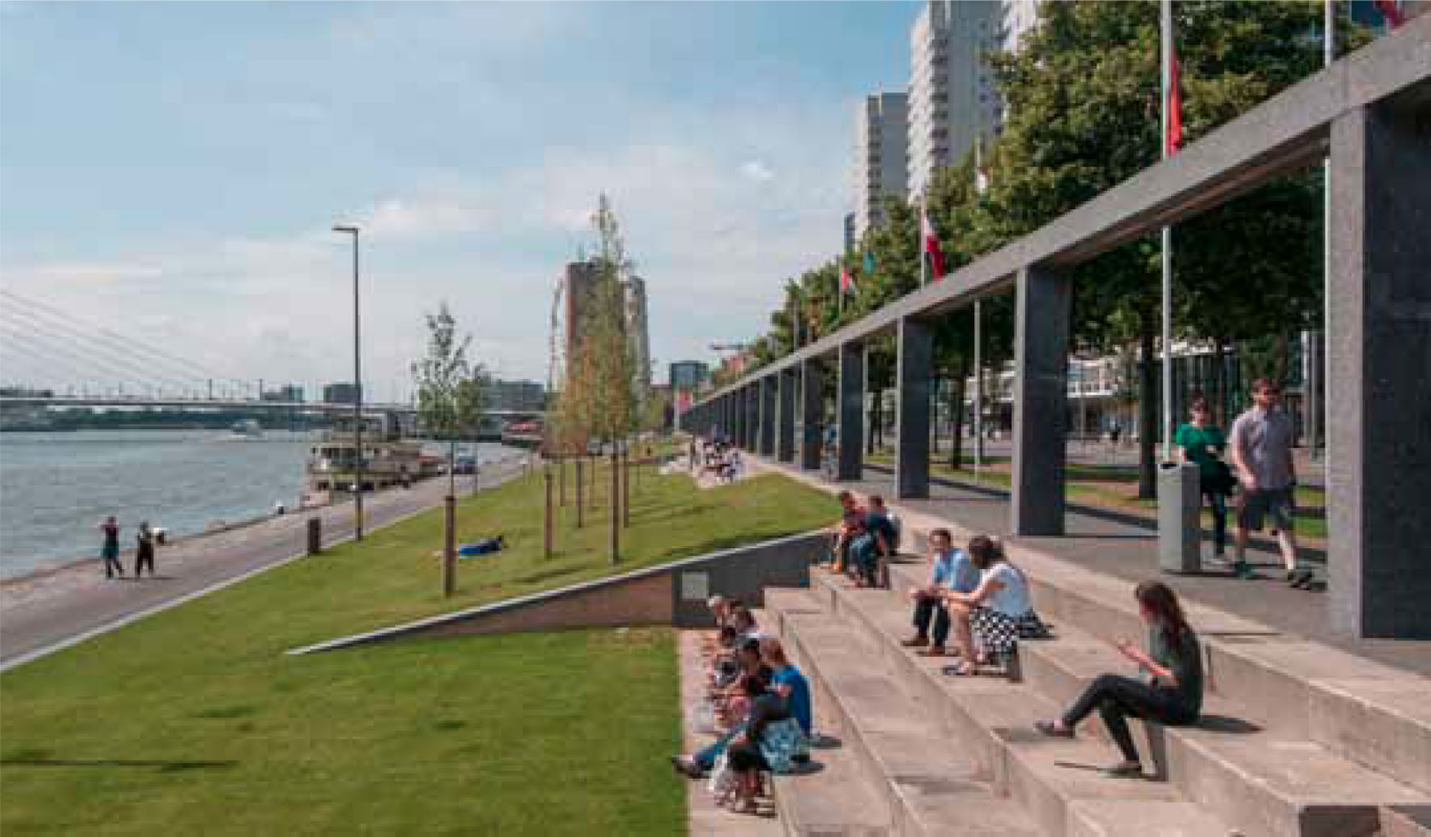
View of Boomjeskade in Rotterdam. The impermeable pavement of the riverbank was replaced with grass creating a soft infrastructure for flood buffering that at the same time serves as a green space for people to use. (Photo: Gemeente Rotterdam 2013; authors’ adaptations and editing)

The Boompjeskade (Fig. 2) is a riverbank location where impermeable pavement has been replaced with grass creating space for water to infiltrate and to be retained as well as space for people to use. It is a pilot site for greening the riverbanks. Such attempts to create soft edges between quays and the river failed in past years. The reason of failing is that the river is used only as a main artery for shipping with stringent safety regulations not allowing “greening.” Efforts to “green” unused harbor inlets remain still limited.

Strategies where water bodies and green areas are understood and dealt as interdependent ecosystems can lead to more robust urban designs. Greening of riverbanks is seen as a promising alternative when considering higher risks for flooding (Delta Program 2012). Overall “soft edges” or greening are often considered controversial. This is evinced by the lack of references of supportive ecosystem services in visions, plans, and strategies. This may be partially the result of the limited collaboration between the spatial planning and sustainability offices with the ecology office in solution searching and designing.

**Fig. 3:**
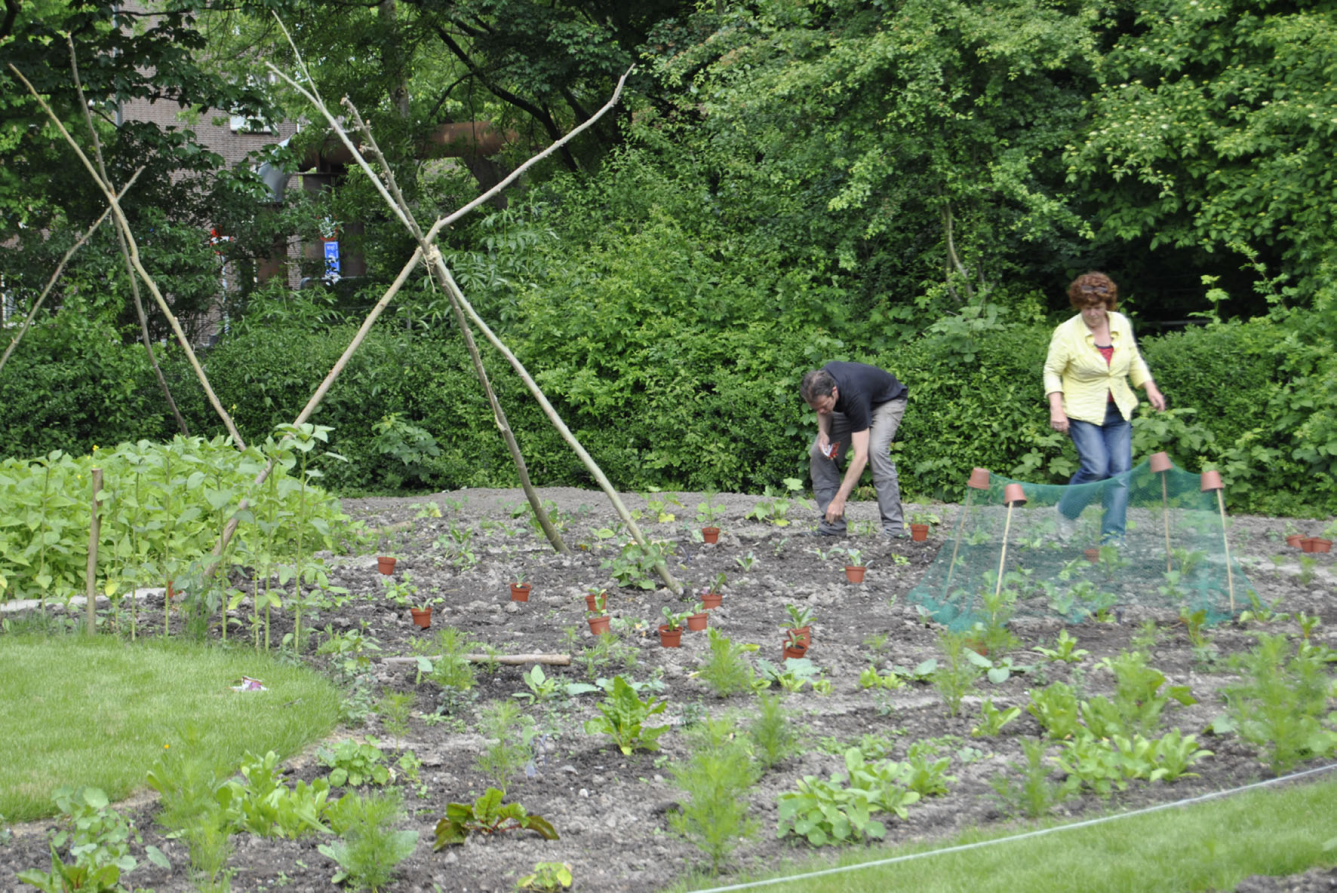
Urban agriculture initiative in Rotterdam city (Photo: authors, June 2013)

(b) *There is lack of a holistic approach to consider all aspects of urban ecosystems and environmental quality at city scale.* There is a lack of a holistic approach that will capture the synergies and interdependencies of all city elements: red infrastructure (buildings and critical infrastructure), green infrastructure (parks, trees, green walls and roofs, and urban agriculture spaces), and blue infrastructure (the rivers and the waterfront). This finding is supported by the lack of reference of provisioning ecosystem services in policy plans and strategies with a city-scale focus (see Table S1 in the Supplementary Material). A new holistic approach to ecological urban planning needs to not only capture the broad and complex urban ecosystem characteristics, but also to be a basis for a new understanding about urban ecosystems and to form a new discourse for urban planning at large. As a result, this creates a need to have an overarching understanding of the developments and of the demands for environmental quality at a city scale.

(c) *There is a disconnection between long*-*term vision, and short*-* and medium*-*term action in projects.* Planning officers expressed that there was a “fatigue” of too many unconnected visions created in the past. This resulted in an aversion to overarching visions and in an interest on how visions can directly relate to improving the city via specific projects. With this tendency to focus on action, there is a risk of losing the trail from the vision to the action when there are no trail keepers in the city’s planning process that remain to their position on the long term. The different projects that bring to life the visions that have been formulated have a project-management horizon. As thus, it is often the case that there is limited ownership of some of these projects due to project teams assembled on project basis. This results in diminishing knowledge on how each project relates to the overall vision for the city.

(d) *There is a need for new ways to engage with citizens and ensure participation in planning*. Although there is increased participation in surveys and in specific planning projects, there is a need to rethink the way participation is organized in order to ensure that citizens are actively involved in planning of their city. Currently the objectives and methods for participation are limited to neighborhoods. There is an increasing interest in engaging with citizens for planning agendas from neighborhood scale to citywide scale, or simply, across scales. At the same time there is a need of new ways to engage with initiatives and to consider innovations by citizens about public space and greening as complementary to city’s planning rather than constraining or obscuring. For example, the booming urban agriculture initiatives in Rotterdam have received attention from the city administration (Fig. 3). However, there is no institutionalized way to assess the needs and motives of citizens who self-organize for urban agriculture and how to tap into the creativity and commitment of these citizens to learn about restoring urban green in neighborhoods.

#### Operational Governance Level

The operational governance level maps the processes and activities that concern implementing and managing policy action plans, infrastructure plans, and assets. On this level, we position the planning practices that relate to “bringing-the-visions-to-the-ground.” The *underlying challenges at the operational governance level that relate to the way policy and programs about ecological features in Rotterdam city are implemented* pointed at the following interrelated challenges: (a) Synergies between planned (before putting on implementation) and ongoing measures are not exploited due to limited information sharing and coordination; (b) There is a need for planning guidelines to designate green areas based on the benefits that can be received from the different types of green; (c) There is no effective strategy on how to scale successful examples of greening in Rotterdam to other locations in the city. These challenges will be elaborated below.

**Fig. 4:**
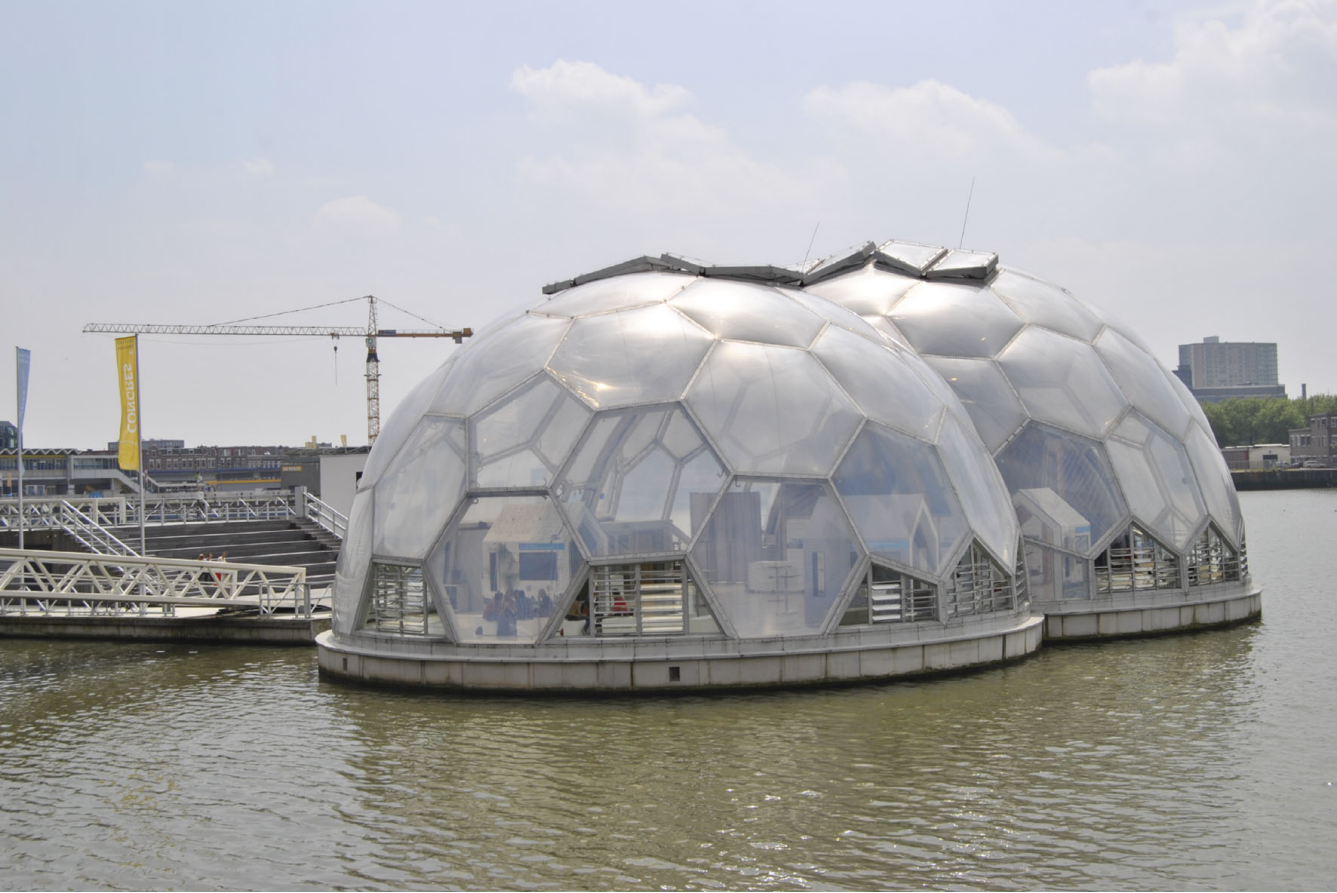
Floating pavilion in Rotterdam’s city ports. An experiment of floating urbanization (Photo: Authors, June 2013)

(a) *Synergies between planned (before putting on implementation) and on*-*going measures are not exploited due to limited information sharing and coordination*. Rotterdam’s planning practice can be characterized as proactive in terms of taking on board early warnings and research outputs about climate adaptation and mitigation as well as considering environmental quality improvements. There is a plethora of plans about climate change adaptation (e.g., floating pavilion, Fig. 4), urban green planning (e.g., restoring green riparian areas), restoration of neighborhood areas into more welcoming and green spaces (e.g., placing trees and green lawns on top of sealed squares), as well as a sustainability agenda and monitoring scheme of the city and its districts. Ongoing plans and strategies are disconnected and synergies between activities and in situ plans are neither addressed nor exploited. The reason is voiced to be the lack of information sharing between the different departmental teams within the city administration that further hinders coordination.

Coordination is currently realized either by assembling inter-departmental teams to work together on a specific project or by informal networks based on personal connections across different city’s departments that are formed at an ad-hoc way to build on the different expertises of officers in order to realize a planning task or project. It thus becomes difficult to build upon the lessons learned and the experience gained from the collaboration realized both at ad-hoc basis and at project-based teams given that these are temporary arrangements. This control-minded approach for collaboration has not been sustainable since it enforces and promotes collaboration only at a project base rather than as a common working practice. An example of fruitful and efficient collaboration between different departments of the city refers to the vision process and strategy development of the City’s Port area. Not every program or redevelopment issue in the city has, however, adopted that good practice.

In the same vein, there is lack of exploiting synergies between policies and plans that address environmental quality and overall sustainability. An example of such weak links is the unexploited relation between linking green space plans and development strategy with city plans on urban health and on improving living conditions of an aging population.

(b) *There is a need for planning guidelines for the designation of green areas based on the benefits that can be received from the different types of green*. There is a need to complement the green design guidelines with planning guidelines that provide strategic information about the multiple benefits of different green and blue elements. If the multiple ecosystem benefits are recognized, planners may be able to make informed choices about the type of elements that can fit and benefit the designated areas when restoring urban ecological features. There is, however, a trade-off about restoring small areas into green spaces: having target to ensure more space for water retention in order to keep the city flood-proofed, there is a competition of space. Space can be used either for green or for water retention. The areas that if “turned” green can retain water are not always (easily) accessible to citizens and as such have less social value. At the same time, the locations with great social potential if restored into green areas do not yield water retention benefits due to their location. As such, there are few locations that these two objectives can meet (e.g., using green space as a water retention area) with the majority of small-scale locations to be competition spaces for implementing climate adaptation measures or urban sustainability measures (e.g., multifunctional spaces to meet multiple socio-ecological needs).

(c) *There is no effective strategy on how to scale successful examples of greening in Rotterdam to other locations in the city.* There is a plethora of experiments in Rotterdam city either directed by policy or emergent such as citizens’ initiatives for greening (including but not limited to urban agriculture). Pilots of introducing soft infrastructures for flood buffering and climate mitigation, such as the greening of walls, the greening of the river banks in Boompjes promenade show how to better integrate green with protective infrastructure measures. Currently Rotterdam experiences a boom in initiatives considering greening and especially of urban agriculture. Efforts to stimulate initiatives from citizens are recently under the scope of policy and planning but are limited to urban farming. Despite the positive attitude of the city’s administration about and toward these initiatives, there is a need to prepare for enabling the scale-up of successful initiatives either in terms of size or in terms of replication of good practices in other areas in the city. A strategy or a benchmark on how to evaluate the benefits of these pilots and initiatives so as to devise mechanisms for supporting them is lacking.

#### Reflexive Governance Level

The reflexive governance level maps the processes and activities the concern monitoring, assessing, and evaluating existing policies considering multiple impacts (social, environmental, ecological, and economic). We identified two *underlying challenges that relate to the way policy and programs about ecological features in Rotterdam city are monitored and evaluated* and concern the evaluation approach of implemented policies. First, policy impacts are assessed against objectives and target levels that are clearly stated in strategic plans and strategies that deliver multiple benefits are not examined. This results in generating new strategies that can perform better toward the same goals without investigating strategies that can deliver multiple benefits. Second, there is limited assessment of social needs in strategic objectives and targets, and as such designed policies may incorporate higher uncertainty for their effective implementation.

## Discussion

### Policy Blind Spots in Urban Ecosystem Governance in Rotterdam

Rotterdam city has an adaptive approach for strategic governance activities in face of the climate change pressures and socio-economic uncertainties. The majority of the vision and strategy documents address climate change pressure to be the top priority for searching ways to ensure resilience. Our analysis shows that the policy attention lies at the following objectives: climate resilience, energy security, and attractiveness for businesses. These objectives remain at the top of the policy agendas and concentrate the majority of plans and visions.

A second tier of objectives concern the fulfillment of the priority objectives and are indirectly related to them: first, the maintenance of good quality and conservation of existing green areas in the city; and second, the improvement of sustainability in the inner city resulting from densification and greening. The greening pathway relates to small-scale (neighborhood, place-specific) introduction of green features in the inner city without proposing land-use changes that may threaten current business activities.

Taking an ecosystem services approach to analyze the governance context, we observe that the strategies and plans focus on the provisioning of regulating ecosystem services. This finding is consistent with the raised policy attention to climate change pressures and how to safeguard the city in the future. However, the majority of the critical visions, strategies, and plans address explicitly all the cultural ecosystem services, whereas there is a blind spot on provisioning and supporting ecosystem services (Table S1 in Supplementary Material). This blind spot is manifested in the current planning approach that does not consider urban ecosystems as interdependent ecological features but as “scapes” of the city that are “planned and planted.” The provisioning of ecosystem services, and especially food provision, is very recently under the policy attention given the booming urban agriculture and gardening initiatives in Rotterdam city.

Last but not the least, the ecosystem services that are under the attention of policy practitioners relate to desirable objectives that are considered during policy formulation. The same desirable objectives are not considered in the negotiation processes for policy implementation creating a mismatch between what is desirable to be achieved (objectives) and what is programmed to be achieved (implementation). This fact shows that there is a mismatch between considering and “designing” based on ecosystem services or discontinuity of the use of ecosystem services framing along the policy cycle (Hauck et al. 2013).

### Dynamics at Each Governance Level and Interdependencies

It is not surprising that the ecosystem services under policy attention are in line with the (policy) objectives that need to be “satisfied” in order to ensure climate proofing of the city of Rotterdam. New visions, policies, and plans adapt and update existing or ongoing measures that provision the same ecosystem services as the existing policies without integrating new ecosystem services in the objectives’ mix. This manifests the workings of a policy renewal cycle that is evinced at the strategic governance level.

The policy renewal cycle is supported by two reinforcing mechanisms: the adaptive policy making approach and the capacity building of policy officers over the past years. The local administration has actively adopted an adaptive policy making approach. It implies that policy measures are regularly evaluated by clearly formulated objectives and adapted over time in order to achieve agreed upon target levels. Whereas the actions and measures are under continuous adaptation and adjustment, desirable policy objectives remain unchanged (Kenny and Meadowcroft 1999). This further enforces the policy renewal cycle and conserves the policy focus on regulating ecosystem services. This adaptive policy process at place explains why there is a limitation and/or delay in considering additional ecosystem services in the planning approach of the city.

The policy renewal cycle is further reinforced by the dominance of specific city’s departments in advocating policy ideas and programs. The dominance of those departments can be assigned to their developing knowledge capacity and expertise that equipped the officers with a good understanding of climate uncertainties and their implications, with the privilege to engage actively with researchers and participate in knowledge networks and partnerships with other cities. The acquired capacity of these officers strengthens their influence on proposing and advocating issues (and the respective ecosystem services) that require policy attention (McAllister et al. 2014). In this way, they influence “policy making by creating institutional routines and procedures that can force decision-making in particular directions” (Howlett et al. 2009, p. 200).

At tactical governance level, specialized approaches in every city’s office create islands of knowledge and result in fragmented understanding of expected and evinced impact of policies and plans. This results in persisting policy disintegration. Disintegration is a wicked problem long entrenched in local government and feeds in the tension between the demand for policy specialization and policy integration. Specialization of city officers is important given the mounting climate change pressures and the socio-cultural dynamics due to the multicultural citizenry. At the same time, policy integration is a pillar for achieving sustainable development and preventing environmental degradation (Collier 1997; Lafferty and Hovden 2003). At the city scale, integration of objectives across different policy and planning offices may result in better solution searching approaches. In Rotterdam, there is evidence that policy integration is at its experimental stage with two programs showcasing it: the Sustainability Vision of 2012 (Gemeente Rotterdam 2012a, b) that attempts to integrate energy efficiency objectives with green space objectives and the Delta Program Strategy that integrates urban planning with flood defense objectives and policies. For the former, the Architectural Biennale acted as a window of opportunity for the integration (Weber 2010). For the latter, the policy platform of the Delta Program created an enabling institutional context for such integration (Persson 2006; Kidd 2007).

At operational governance level there is a diversity of activities (pilots) and initiatives by self-organized citizens and emerging networks. This variety of actions requires new ways of facilitation and coordination that builds on understanding of their motives and of the benefits they bring in.

## Conclusion

Existing knowledge of local government has safeguarded the city with adaptive capacity manifested in policy renewal cycles that give space to new policies, measures, and experiments for adapting to climatic uncertainties and prepare for the risks. These policy renewal cycles lock-in around dominant ecosystem services that are strengthened with new policies ensuring their provisioning. This policy renewal results in a “closed-policy-system” that does not allow additional ecosystem services to be (easily) considered. Such policy renewal cycles are also diagnosed in Finnish governance context by Primmer and Furman (2012, p. 88) that note

It is possible that dominating uses of certain ecosystems, managed under sector-specific administrative structures, are further justified by the broader rhetoric of ecosystem service related opportunities that come with continued use. This kind of a strategy of rhetoric assuring coupled with little change in concrete action has been identified in other areas of natural resource and environmental policy both among private sector actors and in public policy.

Experiments with green infrastructures remain isolated. The lessons learnt are coupled with experiences from initiatives such as urban agriculture. They create innovation capital of the city contributing to the evolution of urban governance. The city’s planning departments are well equipped. More knowledge and processes to interact with citizens who take up initiatives to green the city are however needed in order to build on existing knowledge and expertise, and to facilitate a step-wise broadening of the agenda to new meanings and approaches that capture the multiple benefits of urban ecosystems.

## Electronic supplementary material

Below is the link to the electronic supplementary material.

Supplementary material 1 (PDF 105 kb)
