# Supplementary material for: The Dynamics of Urban Ecosystem Governance in Rotterdam, The Netherlands
Source: Ambio. 2014 Apr 17;43(4):542–55. doi: 10.1007/s13280-014-0512-0 (PMC3989510; doi:10.1007/s13280-014-0512-0)
Supplement: Supplementary file 1 — Supplementary material 1 (PDF 105 kb) [file 13280_2014_512_MOESM1_ESM.pdf]

AMBIO

Electronic Supplementary Material

The Dynamics of Urban Ecosystem Governance in Rotterdam, the Netherlands

Niki Frantzeskaki, Nico Tilie

Detailed description of methodology approach

Given that our research is qualitative, we have used three phases to triangulate our findings: (a) Data collection, including grey literature such as plans, policies and visions of the Rotterdam city and in-person interviews with planners, practitioners and experts; (b) Data analysis, including a governance context analysis and ecosystem services mapping in existing policy and planning documents; and (c) Data validation, realized by facilitated and planned participatory sessions with stakeholders.

Research Methodology Phases

Data Collection	Data Analysis	Data Validation
Methods	Methods	Methods
- Desk research – grey literature review<br>- Field research – in-person interviews with 28 practitioners	- Context analysis – adopting the multi-level governance framework (Loorbach 2010; Frantzeskaki et al. 2014)<br>- Concept-narrative analysis (Arthur 2002)– analysis of policy and planning documents to identify how ecosystem services are referred (TEEB, 2011)	- Participatory workshop – 4-hours-long facilitated discussion around current governance challenges with 17 practitioners and policy officers

Fig. Research Methodology including three phases.

Data Collection Phase

We systematically reviewed vision documents, policies and plans and conducted 28 in-person interviews with planners involved in urban biodiversity and ecosystem management in the city of Rotterdam (from September 2011 until March 2013) in order to integrate their tacit knowledge in examining the governance dynamics.

The interviewees were selected based on three criteria: (a) their knowledge and experience in managing, drafting, implementing and monitoring policy and plans for urban ecosystems and/or urban sustainability plans with a strong link to green spaces and the rivers. Interviewees had more than five years of working experience within Rotterdam's planning system, and some of them had drafted the existing policy and plans for urban green spaces and urban green visions. Interviewees were selected to posit at different governance levels so as to include practices and processes between different governance levels (b) their knowledge about and/or involvement in green initiatives and pilots that relate to restoring or conserving green spaces and urban agriculture in Rotterdam city and (c) their interest in improving the urban environment and in learning from each other and from working together with scientists. Each interview lasted 90 minutes, it was semi-structured and was realized with a set of 24 questions. The analysis of the interviews revealed the degree of policy attention that different issues received and mapped with the frame of ecosystem services.

Data Analysis Phase

We employed two conceptual frameworks to analyze the data and address the research question: (a) a governance context analysis was realized with the aim to examine the governance processes and practices. For the governance context analysis we employed the multi-level governance framework, and (b) for examining the governance attention to ecosystem services we used the TEEB 2011 frame.

In the first step, the governance context will be analyzed by applying the multi-level governance framework that focuses on activities and processes across four levels of governance organization (Loorbach 2010; Frantzeskaki et al. 2014): (a) strategic level including processes and activities of setting long-term goals, policy development, planning, vision, values, identity, culture of the city; (b) tactical level including designing steering activities, programs, funding, establishment of networks and/or partnerships; (c) operational level including implementing and managing policy action plans, infrastructure plans and assets, and (d) reflexive level with monitoring, assessing and evaluating existing policies and assets and their interaction with citizens. For diagnosing the type of activities (strategic, tactical, operational and reflexive) we need to understand and map the relations between different local government departments (and/or offices) within and across the multiple governance levels.

The second step in the analysis was to contrast the challenges with the policy attention to identify policy blind spots in the current state of biodiversity governance in Rotterdam. We reviewed all the policy plans, vision documents, strategy and action implementation plans of the city of Rotterdam across three different offices: urban planning office, sustainability planning office and climate change office. We used concept-narrative analysis in every document to map whether, explicitly or implicitly, there is a reference in terms of action or objective setting on the ways ecosystem services can be provided and safeguarded. In this way we examine which ecosystem services receive attention either in policy formulation and/or in policy design and implementation that will later inform about possible integration points for a holistic strategic program about urban ecosystems.

Table Mapping of ecosystem services that are addressed in core vision and program documents.

	Landmark Visions and Programs							
Ecosystem Services	P-2005a	S-2007a	S-2007b	S-2007c	S-2009a	P-2013a	P-2012a	S-2012
PROVISIONING								
Food								
Raw Materials								
Fresh Water								
Medical Resources								
REGULATING								
Climate regulation		1					1	1
Air filtering	1						1	1
Carbon sequestration								1
Flood protection	1	1					1	
Heat island prevention/mitigation							1	1
Wastewater treatment								
Soil erosion prevention	1							
Maintenance of soil fertility								
Maintenance of healthy soils								
Pollination								
Seed dispersal								

Pest regulation								
Noise regulation			1	1			1	
SUPPORTING								
Habitat for Species	1							
Maintenance of genetic diversity								
CULTURAL								
Recreation	1	1	1	1		1		
Tourism						1		
Aesthetic appreciation	1	1	1	1	1	1	1	1
Cultural, artistic inspiration	1		1	1				
Spiritual value								
Sense of place (identity)	1	1	1	1	1	1		1

Note 1: 1 Indicates explicit reference to the ecosystem service either in the concept of benefit or function either as part of the narrative or even more explicit as an objective or expected benefit from the implementation of a policy. Note 2: With “S” we refer to Visions and with “P” we refer to planning program. Note 3: With grey we mapped the vision and program from the sustainability planning office and with white we mapped the vision and programs from the spatial planning and development office.

Reference List:

S-2007a: Gemeente Rotterdam (2007a), Rotterdam Urban Vision, Spatial Development Strategy

S-2007b: Gemeente Rotterdam (2007b), Visie Openbare Ruimte Centrum Rotterdam.

S-2007c: Gemeente Rotterdam, (2007c), Verboden Stad - Visie Openbare Ruimte Binnenstad Rotterdam.

S- 2009a: Gemeente Rotterdam (2009), Rotterdamse Stijl, Bomenstructuurvisie.

S-2012: Gemeente Rotterdam (2012), Rotterdam – People make the inner city, Issued on the occasion of the 5th International Architecture Biennale Rotterdam.

P-2005: Gemeente Rotterdam, (2005), Groenplan Rotterdam.

P-2012a: Gemeente Rotterdam, (2012a), Programma Duurzaam, Investeren in duuzaame groei.

P-2013a: Gemeente Rotterdam, (2013), Uitwerking visie openbare ruimte Binnenstad, Groenplan Binnenstad

Validation Phase

The outputs of from the policy context analysis have been validated in a 4-hours long participatory workshop attended by 17 stakeholders including urban planners, green NGOs' representatives and environmental science researchers that were invited in an open dialogue workshop about the future of Rotterdam's biodiversity. The participatory workshop was realized in April 2013 in Rotterdam city. During the workshop the outputs of the policy context analysis have been presented and participants discussed openly each challenge and the underlying processes that define and establish it. The facilitator of the workshop did not add content to the discussions, aimed at prompting the dialogue to reveal the processes behind the challenges and for participants to reflect and question the current practices and planning processes. At the end of the workshop, the notes taken and the recordings were analyzed and reported back to the participants. The presented challenges in this paper are the output of this process.
